# *WUSCHEL-related Homeobox* genes in *Populus tomentosa*: diversified expression patterns and a functional similarity in adventitious root formation

**DOI:** 10.1186/1471-2164-15-296

**Published:** 2014-04-21

**Authors:** Bobin Liu, Lin Wang, Jin Zhang, Jianbo Li, Huanquan Zheng, Jun Chen, Mengzhu Lu

**Affiliations:** 1 Key Laboratory of Forest Genetics & Biotechnology of Ministry of Education, Nanjing Forestry University, Nanjing 210 037, China; 2 State Key Laboratory of Tree Genetics and Breeding, Research Institute of Forestry, Chinese Academy of Forestry, Beijing 100 091, China; 3 Key Laboratory of Non-wood Forest Product of State Forestry Administration, School of Forestry, Central South University of Forestry and Technology, Changsha 410 004, China; 4 Department of Biology, McGill University, 1205 Dr Penfield Avenue, Montreal, Quebec H3A 1B1, Canada

**Keywords:** Adventitious root, Expression, Homeobox, *Populus*, WOX, Wuschel-related

## Abstract

**Background:**

WUSCHEL (WUS)-related homeobox (WOX) protein family members play important roles in the maintenance and proliferation of the stem cell niche in the shoot apical meristem (SAM), root apical meristem (RAM), and cambium (CAM). Although the roles of some WOXs in meristematic cell regulation have been well studied in annual plants such as *Arabidopsis* and rice, the expression and function of WOX members in woody plant poplars has not been systematically investigated. Here, we present the identification and comprehensive analysis of the expression and function of WOXs in *Populus tomentosa*.

**Results:**

A genome-wide survey identified 18 WOX encoding sequences in the sequenced genome of *Populus trichocarpa* (*PtrWOXs*). Phylogenetic and gene structure analysis revealed that these 18 *PtrWOXs* fall into modern/WUS, intermediate, and ancient clades, but that the *WOX* genes in *P. trichocarpa* may have expanded differently from the *WOX* genes in *Arabidopsis*. In the *P. trichocarpa* genome, no *WOX* members could be closely classified as *AtWOX3*, *At*WOX6, *AtWOX7*, *AtWOX10*, and *AtWOX14*, but there were two copies of *WOX* genes that could be classified as *PtrWUS*, *PtrWOX2*, *PtrWOX4*, *PtrWOX5*, *PtrWOX8/9*, and *PtrWOX11/12*, and three copies of *WOX* genes that could be classified as *PtrWOX1* and *PtrWOX13*. The use of primers specific for each *PtrWOX* gene allowed the identification and cloning of 18 *WOX* genes from *P. tomentosa* (*PtoWOXs*), a poplar species physiologically close to *P. trichocarpa.* It was found that PtoWOXs and PtrWOXs shared very high amino acid sequence identity, and that PtoWOXs could be classified identically to PtrWOXs. We revealed that the expression patterns of some *PtoWOXs* were different to their *Arabidopsis* counterparts. When *PtoWOX5a* and *PtoWOX11/12a*, as well as *PtoWUSa and PtoWOX4a* were ectopically expressed in transgenic hybrid poplars, the regeneration of adventitious root (AR) was promoted, indicating a functional similarity of these four *WOXs* in AR regeneration.

**Conclusions:**

This is the first attempt towards a systematical analysis of the function of *WOX*s in *P. tomentosa*. A diversified expression, yet functional similarity of *PtoWOX*s in AR regeneration is revealed. Our findings provide useful information for further elucidation of the functions and mechanisms of *WOX*s in the development of poplars.

## Background

Homeobox (HB) proteins were first discovered in *Drosophila*. They are a superfamily of transcriptional factor proteins containing a conserved 60-amino acid homeodomain (HD). HB proteins have been found in all eukaryotic organisms tested [1-3]. In plants, a great number of HD-containing transcriptional factors have been identified in both monocots and dicots [4], with KNOTTED1 being the first identified HD-containing protein [5]. The HB protein superfamily is classified into six families based on HD sequence, location, association with other functional domains, and the protein size and structure. These classifications are: homeodomain-leucine (HD-Zip), plant homeodomain (PHD)-finger, BELL, zinc finger-homeodomain (ZF-HD), WUSCHEL (WUS)-related homeobox (WOX), and KNOTTED1-like-homeobox (KNOX) [4].

The WOX family of HB proteins is distinguished by the phylogenetic relatedness of its homeodomain, and is a plant specific HB transcription factor family [6]. The *Arabidopsis* genome encodes at least 15 WOX proteins, and these are classified into three clades: [7] a modern/WUS clade including WUS and AtWOX1-7; an intermediate clade including AtWOX8, 9, 11, and 12; and an ancient clade containing AtWOX10, 13, and 14 [6,8]. *Arabidopsis* WOXs regulate key developmental processes including stem cell maintenance in the SAM, RAM, and CAM, embryo apical-basal polarity patterning, and lateral organ development [8]. *AtWUS*, a member of the modern WOX clade, is expressed specifically in the organizing center of the SAM, and is required for maintaining stem cells via a feedback loop with CLV3, a peptide ligand that interacts with leucine-rich repeat receptor kinases in neighboring cells to restrict the size of the SAM in *Arabidopsis*[9]. *AtWOX5*, also a member of the modern WOX clade, is expressed in root quiescent centre (QC) cells surrounded by the stem cells [10]. The AtWOX5 protein is essential for stem cell maintenance via a negative feedback signal provided by CLE40 [11]. *AtWOX4* is strongly expressed in the CAM, and regulates vascular stem cell maintenance [12,13]. *AtWOX2* is expressed in zygotes, and is involved in regulating cell fate in the apical and basal lineage of developing embryos [14]. AtWOX3/PRS1 is involved in lateral organ development through recruiting organ founder cells forming the lateral domain in *Arabidopsis*[15]. *AtWOX6* is expressed abundantly in developing ovules, and is involved in either ovule patterning or differentiation [16]. With regards to stem cell maintenance in the SAM and RAM, it is noteworthy that AtWUS and AtWOX5 are interchangeable [10]. Furthermore, the function of AtWOX3 in lateral organ development can be fully complemented by AtWUS [15], and partially complemented by AtWOX4 [17]. Taken together, it appears there is a common mechanism of action among modern WOX proteins. It has recently been demonstrated that members of the modern WOX clade have evolved, through the acquisition of a conserved WUS-box, a repressive activity important for leaf blade outgrowth regulation [8].

For the intermediate *WOXs*, *AtWOX8*, co-expressed with *AtWOX2* in the zygote, is required for normal development of the pre-embryo [14]. Similar to *AtWOX8* in *Arabidopsis*, *PaWOX8*/*9* is highly expressed at the early zygotic growth stages and the later embryo stages in *Picea abies*[18]. The *AtWOX9* gene is involved in maintaining cell division and preventing premature differentiation in the *Arabidopsis* SAM [19]. The expression patterns and function of *AtWOX11* and *12* are currently unknown in *Arabidopsis*, but *OsWOX11* in rice is reported to be required for activating shoot-borne root development by directly repressing *RR2*, a cytokinin type-A responsive regulator gene [20].

With regards to the most conserved ancient plant WOX proteins, the expression and function of *AtWOX10* is unknown. Although *AtWOX13* is expressed ubiquitously it is most strongly expressed in developed flowers and young siliques, where it is involved in the promotion of replum formation during fruit development [21]. *AtWOX14* is thought to prevent premature differentiation of primary roots, lateral roots, and floral organs [22]. *AtWOX14* is predominantly expressed in vascular tissues and acts redundantly with *AtWOX4* in vascular cell differentiation [23].

Poplars are a widely distributed group of economic plants. As a rapid growth species, vegetative growth leads to higher production of plant biomass [24], thus poplars are considered as feedstocks for bioenergy and timber [25,26]. *Populus tomentosa* is a Chinese native poplar species with a high economic value and is widely planted in northern China. Because of the key roles that WOX proteins play in stem cell maintenance and lateral organ development, WOX proteins are potential targets for better and faster growth of *P. tomentosa*. Here, we identify 18 WOX encoding genes in *P. tomentosa* (*PtoWOXs*). We provide a comprehensive analysis of the expression and function of the *PtoWOXs*. We reveal that although members of all three WOX clades exist in *P. tomentosa*, PtoWOXs expanded differently from those of *Arabidopsis*. The expression of *PtoWOXs* was found to differ from their *Arabidopsis* counterparts, with many showing significant expression in the roots, and being inducible in the regeneration of adventitious roots (ARs). Furthermore, we report that the ectopic expression of *PtoWOXs* from both modern and intermediate WOX clades promotes the regeneration of ARs.

## Results

### Identification and phylogenetic analysis of *WOX* genes in *Populus trichocarpa* and *P. tomentosa*

**Figure 1:**
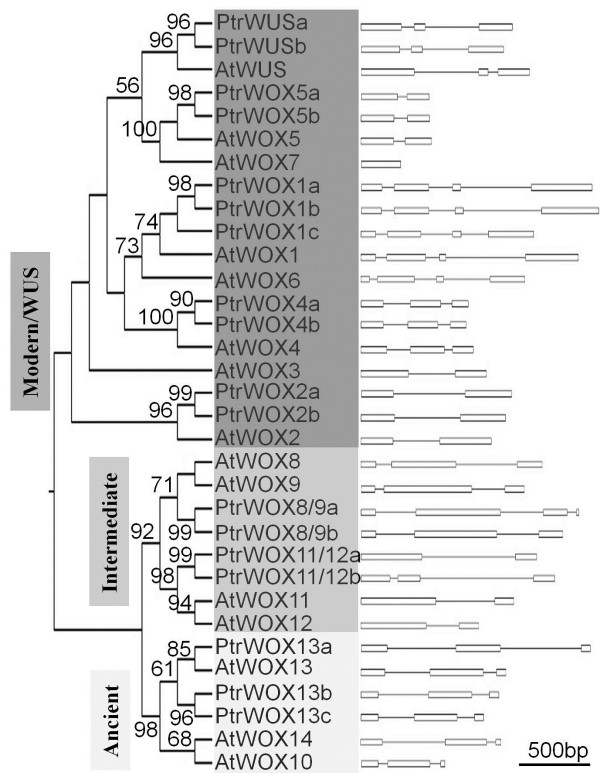
**Phylogenetic tree of WOX proteins and gene structure of corresponding *WOX *genes from *Populus trichocarpa *and *Arabidopsis thaliana*.** Deduced full-length amino acid sequences were aligned using ClustalX 2.0. The phylogenetic tree was constructed using phyML and the maximum likelihood method. The three clades (ancient, intermediate, and modern/WUS) are indicated as different grayscale boxes in the left panel. Empty boxes and black lines in the right panel represent exons and introns respectively. Support values are shown on selected branches.

The *P. tomentosa* genome has not been sequenced. Therefore, to initiate functional analysis of WOXs in *P. tomentosa* we searched for *WOX* genes in the sequenced genome of *P. trichocarpa*, a poplar species physiologically close to *P. tomentosa*. We used all 15 known *Arabidopsis* WOX protein sequences as queries to blast against the *P. trichocarpa* genome database. Eighteen *WOX* gene sequences were identified in the *P. trichocarpa* genome; this is similar to the number of *WOXs* found in *Arabidopsis* (15) [7], *Oryza sativa* (13) [27], *Zea mays* (18) [28], and *Vitis vinifera* (12) [28]. The putative WOX proteins in *P. trichocarpa* (PtrWOXs) ranged from 171 to 390 amino acids in length (Table 1). A phylogenetic tree of WOX proteins from *P. trichocarpa* and *Arabidopsis* was generated based on the full WOX amino acid sequences (Figure 1, left side). Similar to the HD sequence based phylogeny of known plant WOX proteins [29], the 18 PtrWOXs fell into three major branches: the modern/WUS clade, the intermediate clade, and the ancient clade; these were similar to their *Arabidopsis* counterparts (Figure 1, left). The modern/WUS clade contained 11 PtrWOXs; these could be divided into five subclasses based on their relationship with their *Arabidopsis* counterparts. These were named as PtrWUS, PtrWOX1, PtrWOX2, PtrWOX4, and PtrWOX5. The intermediate clade consisted of four PtrWOXs; these were classified into two subclasses: PtrWOX8/9, and PtrWOX11/12. The ancient clade contained only one subclass: PtrWOX13. This classification was further confirmed by the exon/intron organization of the WOX coding sequences in *P. trichocarpa* and *Arabidposis* (Figure 1, right). With the exception of *PtrWOX8/9a* and *PtrWOX11/12b*, all other *PtrWOX* genes shared a similar intron/exon organization in the size and arrangement of introns and exons with their corresponding *AtWOX* counterparts.

**Table 1:** **Summary of the *PtrWOX *** **gene family**

**Gene name**	**Locus**	**Genomic position**	***Arabidopsis WOXs*** *	**Length**	**Size (kD)**
*PtrWUSa*	Potri.005G114700	Chr05: 8858336-8859980	*AT2G17950*	264	30.004
*PtrWUSb*	Potri.007G012100	Chr07: 958955-960287	*AT2G17950*	264	29.274
*PtrWOX1a*	Potri.012G047700	Chr12: 4446344-4449088	*AT3G18010*	387	43.851
*PtrWOX1b*	Potri.015G039100	Chr15: 3566414-3569217	*AT3G18010*	374	42.326
*PtrWOX1c*	Potri.010G111400	Chr10: 13009243-13010950	*AT3G18010*	316	35.699
*PtrWOX2a*	Potri.001G237900	Chr01: 24918823-24920230	*AT5G59340*	245	27.877
*PtrWOX2b*	Potri.009G029200	Chr09: 3976470–3977823	*AT5G59340*	246	27.601
*PtrWOX4a*	Potri.002G124100	Chr02: 9309765-9311320	*AT1G46480*	213	24.417
*PtrWOX4b*	Potri.014G025300	Chr14: 2169818-2171379	*AT1G46480*	213	24.463
*PtrWOX5a*	Potri.008G065400	Chr08: 3963469-3964104	*AT3G11260*	181	21.009
*PtrWOX5b*	Potri.010G192100	Chr10: 18688337-18689050	*AT3G11260*	171	19.615
*PtrWOX8/9a*	Potri.004G051600	Chr04: 4008260-4010297	*AT2G33880*	390	43.072
*PtrWOX8/9b*	Potri.011G061400	Chr11: 5466036-5468417	*AT2G33880*	377	41.601
*PtrWOX11/12a*	Potri.013G066900	Chr13: 5239050-5240692	*AT3G03660*	255	28.086
			*AT5G17810*		
*PtrWOX11/12b*	Potri.019G040800	Chr19: 4693429-4695238	*AT3G03660*	226	25.001
			*At5G17810*		
*PtrWOX13a*	Potri.005G101800	Chr05: 7788135-7790958	*AT4G35550*	248	27.984
*PtrWOX13b*	Potri.005G252800	Chr05: 25454469-25456507	*AT4G35550*	216	24.639
*PtrWOX13c*	Potri.002G008800	Chr02: 484202-486345	*AT4G35550*	215	24.479

*Arabidopsis WOX genes that are closely classified with *PtrWOXs* according to the phylogenetic analysis of protein sequences.

All PtrWOXs contained highly conserved HD residues (Additional file 1: Figure S1A). A conserved WUS-box domain (TLXLFP) located downstream of the HD domain [7] was also found in members of the modern clade, but not in the other clades (Additional file 1: Figure S1B). In addition, an EAR-like domain was present in the C-terminal ends of PtrWUSa, PtrWUSb, PtrWOX5a, and PtrWOX5b (Additional file 1: Figure S1C).

Although the 18 PtrWOXs could be classified into modern/WUS clade, intermediate, or ancient clades, it is interesting that PtrWOXs may have expanded differently in *Arabidopsis* (Figure 1 left). Two copies of PtrWOXs could be classified as WUS (PtrWUSa and b), WOX2 (PtrWOX2a and b), WOX4 (PtrWOX4a and b), WOX5 (PtrWOX5a and b), WOX8/9 (PtrWOX8/9a and b), and WOX11/12 (PtrWOX11/12a and b), and three copies of PtrWOXs could be classified as WOX1 (PtrWOX1a, b, and c) and WOX13 (PtrWOX13a, b, and c). However, no PtrWOXs were closely related to AtWOX3, AtWOX6, AtWOX7, AtWOX10, and AtWOX14 in the phylogenetic tree (Figure 1, left). Similarly, close orthologs of AtWOX7, AtWOX10, AtWOX12, and AtWOX14 were missing from *Vitis vinifera*[28], *Picea abies*[30]*,* and other woody plant species [28]. Details of these PtrWOX encoding genes, including their corresponding *Arabidopsis* counterparts, and amino acid lengths are listed in Table 1.

**Figure 2:**
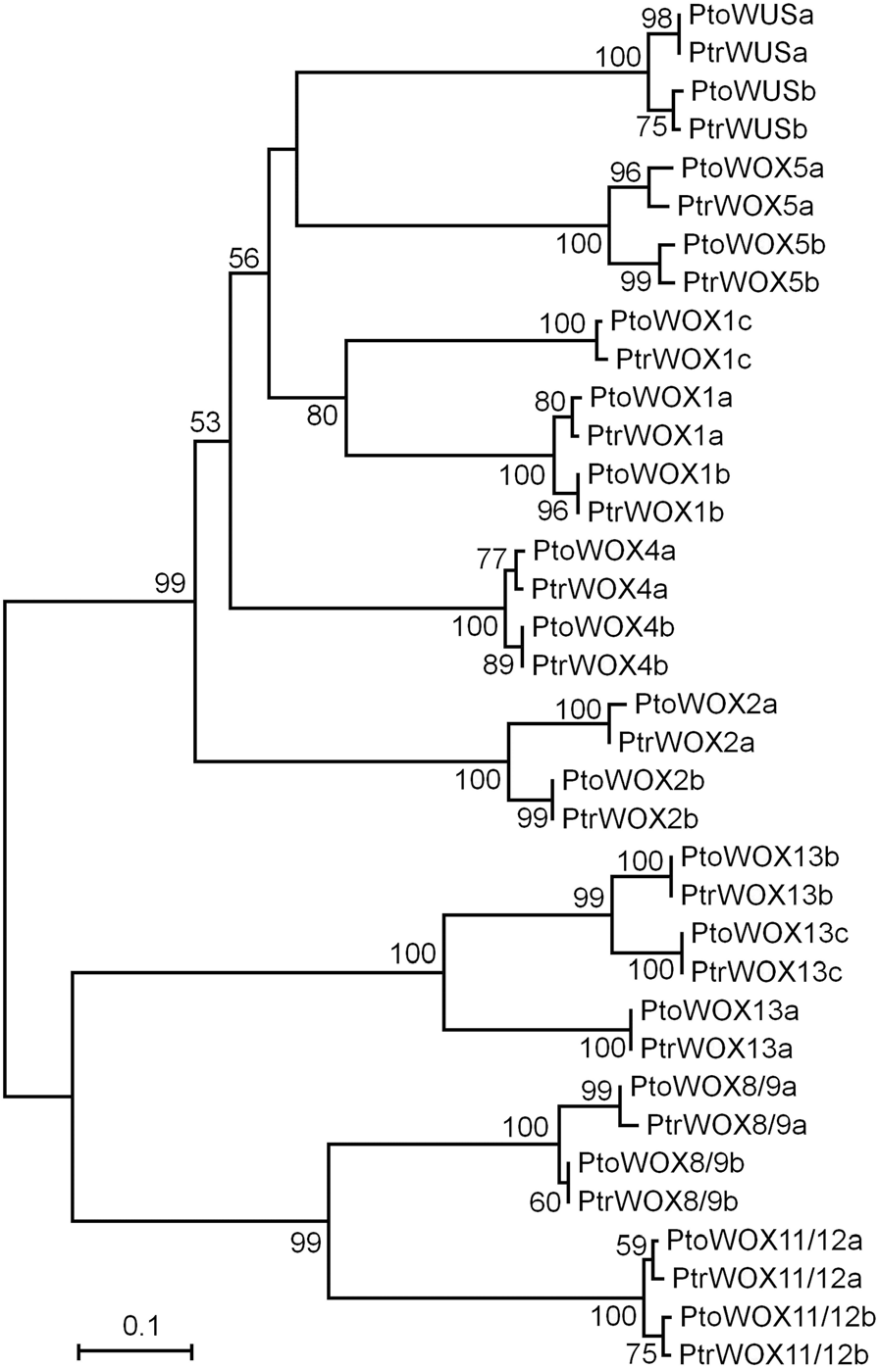
**Phylogenetic tree of WOX family proteins from *P. trichocarpa *and *P. tomentosa*.** Multiple alignment of full-length WOX protein sequences from the two poplar species was executed using Clustal X2.0, and a phylogenetic tree constructed using MEGA 4.0 and the neighbor-joining (NJ) method with 1000 bootstrap replicates. Bootstrap support is indicated at each node.

Using primers specific to each *PtrWOX* gene (Additional file 2: Table S1), we identified and cloned 18 corresponding *WOX* genes from *P. tomentosa* (*PtoWOXs*). We found that PtoWOXs and PtrWOXs shared very high amino acid sequence similarities (86-99%) and CDS sequence identities (Additional file 2: Table S2). Phylogenetic analysis revealed that the PtoWOXs could be classified identically to PtrWOXs (Figure 2).

### Chromosomal location and gene duplication of *WOX* genes in *P. trichocarpa*

To better understand how *WOX* genes expanded in poplars, we took advantage of the sequenced genome of *P. trichocarpa* and mapped the chromosomal location of the 18 *PtrWOX* loci. These 18 *PtrWOX* sequences were unevenly distributed among the 14 chromosomes, with the exception of chromosomes III, VI, XIII, XVI, and XVIII (Additional file 1: Figure S2). The chromosomal duplication map of *P. trichocarpa* was generated previously [31]. We identified five pairs of *PtrWOX* genes, *PtrWOX1a-PtrWOX1b*, *PtrWOX4a-PtrWOX4b*, *PtrWOX5a-PtrWOX5b*, *PtrWOX11/12a-PtrWOX11/12b*, and *PtrWOX13b-PtrWOX13c* in the duplicated segments of the *P. trichocarpa* genome. This suggests that expansion of the *PtrWOX* gene family was, at least partially, caused by chromosomal duplication events during the evolution of poplar species.

### Nuclear localization of PtoWOXs

**Figure 3:**
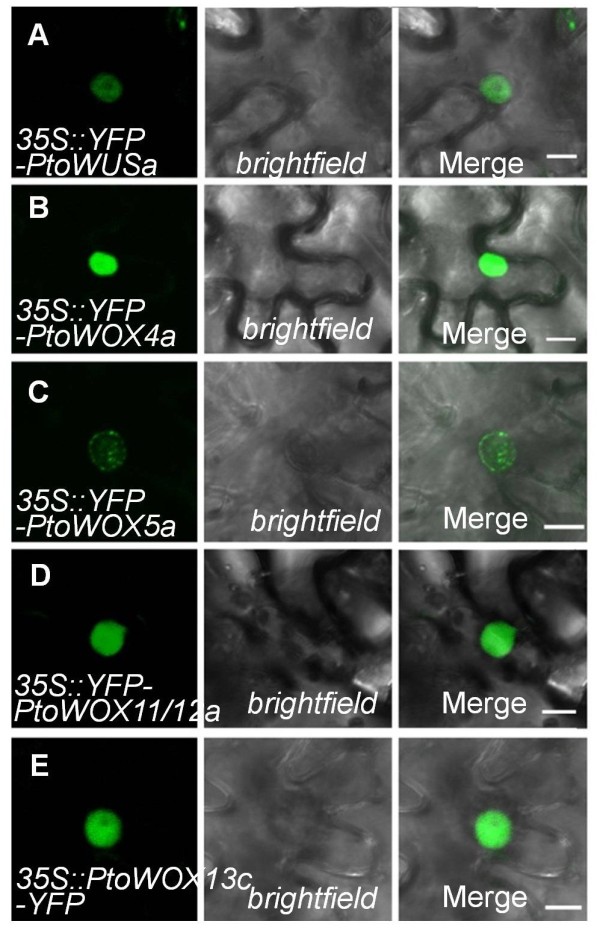
**Subcellular localization of five PtoWOX proteins.** The nuclear localization of PtoWUSa **(A)**, PtoWOX4a **(B)**, PtoWOX5a **(C)**, PtoWOX11/12a **(D)**, and PtoWOX13c **(E)** in a tobacco lower epidermal cell. The YFP channel, the bright field, and the merged images are shown on the left, middle, and right panels respectively.

As a first step towards understanding the functions of WOXs in *P. tomentosa*, we examined the subcellular localization of PtoWUSa, PtoWOX4a, PtoWOX5a, PtoWOX11/12a, and PtoWOX13c members of selected PtoWOX subclasses. As transcription factors, WOXs should be targeted to the nucleus. To test this, we fused YFP at N-termini of PtoWUSa, PtoWOX4a, PtoWOX5a, and PtoWOX11/12a and the C-terminus of PtoWOX13c. In a transient expression assay of *Nicotiana benthamiana* leaf epidermal cells all fusion proteins were found in the nucleus (Figure 3).

### Diversified expression patterns of *PtoWOX* genes

**Figure 4:**
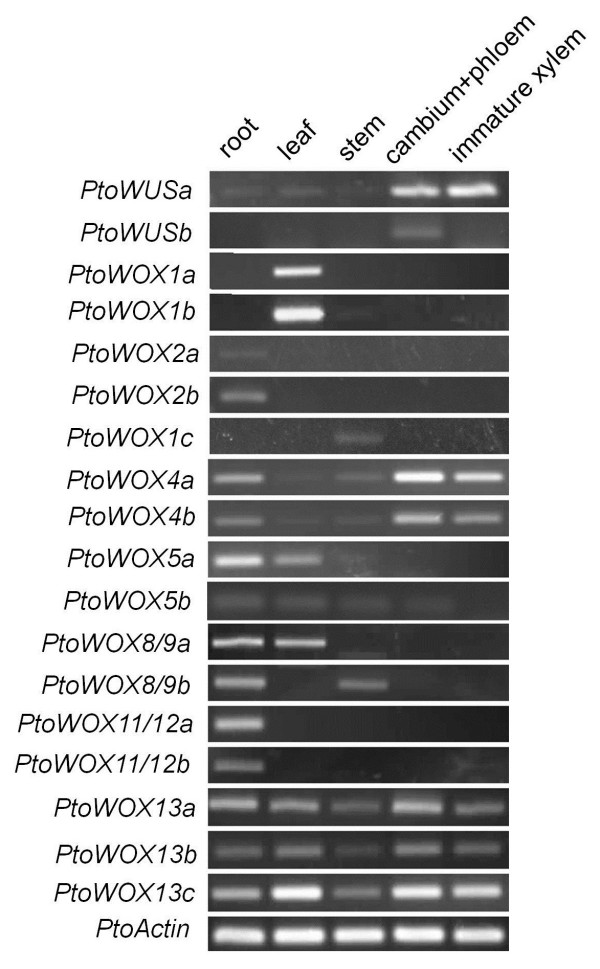
**Semi-quantitative analysis of *P*to*WOX *genes.** The expression of *PtoWOXs* in roots, leaf, stem, CAM zone, and immature xylem from *P. tomentosa* Carr. The amplification cycle used was 32 for *PtoWOX4a*, *4b*, and *13c*; 35 for *PtoWUSa*, *PtoWOX1a*, *13a* and *13b*, and *11/12a* and *11/12b*; 40 for all other *WOXs*, and 30 for the *PtoActin* reference control.

To gain insights into possible developmental and physiological functions of WOX proteins in *P. tomentosa*, the expression pattern of *WOX* genes in *P. tomentosa* Carr. were analyzed by semi-quantitative RT-PCR (Figure 4, Table 2). Two *PtoWOX2s* and two *PtoWOX11/12 s* were mainly expressed in the roots. In contrast, *PtoWUSb,* and three *PtoWOX1s* were nearly absent from roots. Further to this, *PtoWOX1a* and *1b* were only expressed in leaves and *PtoWOX1c* only in the stem. *PtoWOX5a* and *PtoWOX8/9a* were highly expressed in both roots and leaves, while *PtoWOX8/9b* was expressed in both roots and stem. It is noteworthy that *PtoWUSa*, *PtoWUSb*, *PtoWOX4a*, and *PtoWOX4b* were strongly expressed in the CAM zone of poplar stems. In *Arabidopsis*, *AtWUS* is only expressed in the SAM [32]. In addition to the enrichment of *PtoWUSa* and *PtoWUSb* in the CAM zone, *PtoWUSa* was also expressed, albeit weakly, in roots, leaves, and stems. Similarly, *AtWOX5* is restricted to the RAM [10], while *PtoWOX5b* was ubiquitously expressed. The only subclass of the ancient WOX clade in poplars, *PtoWOX13s* (*PtoWOX13a*, *b*, *c*), also exhibited a ubiquitous expression pattern. In a separate research project conducted in our laboratory, we generated a set of RNA-seq data for the hybrid poplar *Populus alba* X *Populus glandulosa*. Quantification of expression of the corresponding 18 *WOX* genes in *P. alba* X *P. glandulosa* (*PagWOXs*; Additional file 1: Figure S3, Additional file 2: Table S3) indicated that the expression pattern of 18 *PagWOX* genes in the hybrid poplar was similar to that of the 18 *PtoWOX* genes in *P. tomentosa*, though there was a discrepancy between *PtoWUSb* and *PagWUSb*.

**Table 2:** **Summary of the expression pattern of *AtWOXs *** **and *PtoWOXs***

***PtoWOXs***	**Expression pattern**	***AtPWOXs***	**Expression pattern**	**Ref**
*PtoWUSa*	Root, leaf, SAM, *cambium*, *xylem*	*AtWUS*	*SAM*	[32]
*PtoWUSb*	*Cambium*, *xylem*			
*PtoWOX1a*	*Leaf*	*AtWOX1*	Between the adaxial and abaxial domains of leaf	[33]
*PtoWOX1b*	*Leaf*			
*PtoWOX1c*	*Stem*			
*PtoWOX2a*	*Root*	*AtWOX2*	Apical embryo domain	[34]
*PtoWOX2b*	*Root*			
No closely classified counterparts		*AtWOX3*	flower primordia, floral organ primordia, and young leaf primordia.	[35]
*PtoWOX4a*	Root, Leaf, stem, *cambium*, *xylem*	*AtWOX4*	*Cambium*, trichomes, stomata, phloem, pericycle	[12]
*PtoWOX4b*				
*PtoWOX5a*	RAM, leaf	*AtWOX5*	RAM	[10]
*PtoWOX5b*	Root, leaf, stem			
No closely classified counterparts		*AtWOX6*	Differentiating primordia and *developing ovules*	[16]
No closely classified counterparts		*AtWOX7*	unknown	
*PtoWOX8/9a*	*Root*, *leaf*	*AtWOX8*	zygote, proembryo and embryo	[14]
		*AtWOX9*	Developing embryos, proliferating tissues, SAM, leaf primordia, floral meristems, epidermal layer of the placenta and growing septum	[19]
*PtoWOX8/9b*	*Root*, *stem*			
No closely classified counterparts		*AtWOX10*	unknow	
*PtoWOX11/12a*	*Root*	*OsWOX11*	Cell division regions in roots and shoots.	[20]
*PtoWOX11/12b*		*AtWOX11*, *12*	unknown	
*PtoWOX13a*	Root, leaf, SAM, *cambium*, *xylem*	*AtWOX13*	Root tip, emerging lateral roots, root, SAM and leave vasculature, *gynoecia*	[21]
*PtoWOX13b*				
*PtoWOX13c*				
No closely classified counterparts		*AtWOX14*	Vascular tissue	[23]

The *WOX* expression patterns in other plant species were published in the corresponding references listed. Tissue names in italic indicate strong expression.

### *Promoter::GUS* based analysis of expression of *PtoWOX* genes

**Figure 5:**
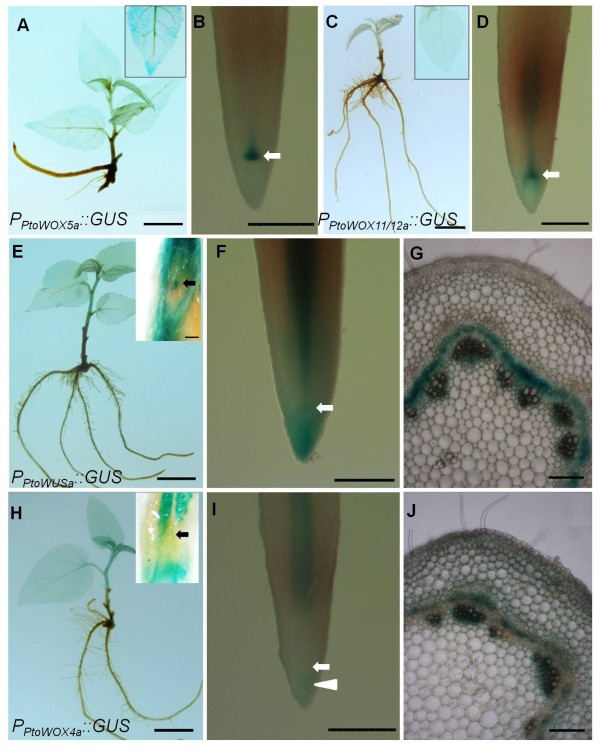
**GUS-staining assays of four *PtWOX *promoters. (A–B)** GUS expression in **(A)** 2-week-old sapling and **(B)** root of *P*_*PtoWOX5a*_*::GUS*-expressing poplar plants. The insert in A shows GUS expression in the leaf, and that the GUS expression in B is mainly focused on a small area immediately behind the root cap in AR tips. **(C–D)** GUS expression in **(C)** 2-week-old sapling and **(D)** root tip of *P*_*PtoWOX11/12a*_*::GUS*-expressing poplar plants. GUS is expressed in a small area immediately behind the root cap in AR tips, and the insert in C shows no GUS expression in leaf. **(E–G)** GUS expression in **(E)** 2-week-old sapling, **(F)** root including a small area behind the root cap indicated by an arrow, and **(G)** the CAM zone of *P*_*PtoWUSa*_*::GUS*-expressing poplar plants. The insert in E shows GUS expression in the SAM area in a halved shoot apical part. **(H–J)** GUS expression in **(H)** 2-week-old sapling, **(I)** root tip and **(J)** stem of *P*_*PtoWOX4a*_*::GUS*-expressing poplar plants. The insert in H shows no GUS expression in the SAM area in a halved shoot apical part. The bar represents 1 cm in **A**, **C**, **E**, and **H**; 1 mm in **D**; 0.5 mm in **B**, **F**, and **I**; and 0.1 mm in **G**, **J**, and the inset of **E**.

RT-PCR is a rapid way to examine tissue specificity of gene expression, but it does not provide a good resolution. Since we are interested in the process of the root growth and wood formation in *Populus tomentosa*, a root-specific *PtoWOX11/12a*, a root-enriched *PtoWOX5a*, and two generally expressed but CAM enriched *PtoWOXs* (*PtoWUSa* and *PtoWOX4a*) were selected for *promoter::GUS* assays to examine their expressions in detail. Due to ease of genetic manipulation, the hybrid poplar *P. alba* X *P. glandulosa*, a close relative of *P. tomentosa*, was chosen for gene transformation. In *Arabidopsis*, *AtWOX5* is expressed specifically in the QC cells in the root tips [10]. Semi-quantitative RT-PCR results revealed that in addition to being strongly expressed in the roots *PtoWOX5a* was weakly expressed in the leaves (Figure 4). The *promoter::GUS* assays indicated that *PtoWOX5a* was mainly expressed in a small area behind the cap region of ARs in the *P*_*PtoWOX5a*_*::GUS* transformed hybrid poplar (Figure 5B, see arrow). The *PtoWOX5a* promoter had a weak activity in the leaves (Figures 4 and 5A). Semi-quantitative RT-PCR identified *PtoWOX11/12a* as a root specific *WOX* gene in *P. tomentosa* (Figure 4). A GUS assay confirmed it was only expressed in the roots, especially in a small region behind the AR cap (Figure 5C and D, see arrow). Based on The semi-quantitative expression analysis revealed *PtoWUSa* and *PtoPWOX4a* were strongly expressed in the CAM zone, but were detectable in other examined tissues (Figure 4). GUS staining (Figure 5E–J) revealed that *PtoWUSa* and *PtoPWOX4a* were both strongly expressed in the CAM zone of stems (Figure 5G and J); they were also weakly expressed in roots (Figure 5F and I) as GUS staining of roots of transgenic lines expressing either *P*_*PtoWUSa*_*::GUS* or *P*_*PtoWOX4a*_*::GUS* occurred after a prolonged period. Interestingly, in the *P*_*PtoWUSa*_*::GUS* lines, a small region behind the root cap could be stained (Figure 5F, arrow), though activity of *P*_*PtoWUSa*_::*GUS* was not limited to this small region in roots. In *P*_*PtoWOX4a*_*::GUS* transgenic lines, the small region behind the root cap region was not stained, but the root cap region of ARs could be stained (Figure 5I, arrowhead).

### Dynamic expression of *PtoWOX*s during the regeneration of adventitious shoots and roots in *P. tomentosa*

**Figure 6:**
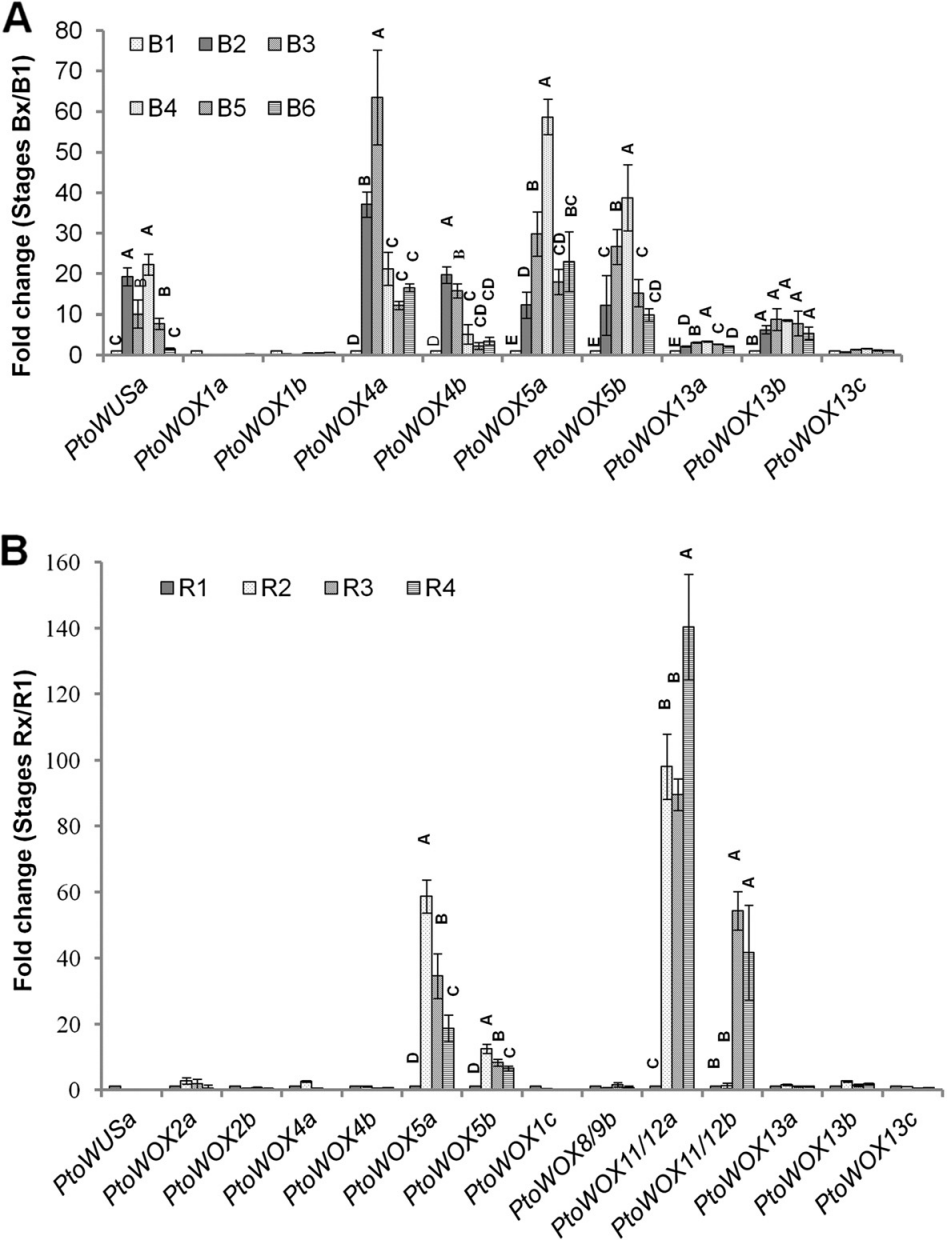
**Quantitative PCR analysis of dynamic expression of *PtoWOXs *in the regeneration of AS and AR. (A)** Dynamic changes of *PtoWOXs* in AS regeneration. The expression of *PtoWUSa*, *PtoWOX4a*, *4b*, *5a*, and *5b* is significantly changed. B1–B6 are six chronological stages during AS regeneration. **(B)** Dynamic changes of *PtoWOXs* in AR regeneration. *PtoWOX5a*, *5b*, *11/12a*, and *11/12b* are significantly changed. R1–R4 are four chronological stages during AR regeneration. Insignificant differences, according to LSD test (*P* < 0.01), are denoted using the same letters. Error bars represent standard deviation.

The regeneration of adventitious shoot (AS) and AR using leaf or stem explants is one of the best tools for rapid propagation and genetic manipulation of poplars. These regeneration processes involve the re-establishment of SAM and RAM, which leads to the final differentiation of leaves and roots. Therefore regeneration of AS and AR is an excellent system for studying stem cell initiation, cell fate determination, and hormonal signaling [36]. AS regeneration is composed of several successive stages, including the B1 pre-induction stage (Additional file 1: Figure S4A), the B2 callus induction stage (Additional file 1: Figure S4B), the B3 callus expansion stage (Additional file 1: Figure S4C), the B4 callus transition stage (Additional file 1: Figure S4D), the B5 AS formation stage (Additional file 1: Figure S4E), and the B6 AS growth stage (Additional file 1: Figure S4F). To investigate whether *PtoWOXs* play roles in the regeneration of AS we examined the dynamics of *PtoWOX* expression using quantitative real-time PCR. Among the 18 *PtoWOXs*, 10 had significant changes in expression during AS regeneration (Figure 6A). The expression levels of *PtoWUSa*, *PtoWOX4a*, *PtoWOX4b*, *PtoWOX5a*, and *PtoWOX5b* changed over twenty-fold during the regeneration of AS. They had different expression dynamics with maximum expression of *PtoWOX4a/b* seen at earlier stages (B2/B3), and *PtoWOX5a/b* at later stages (B4) (Figure 6). All three *PtoWOX13s* were expressed at a relatively low level in AS regeneration but were slightly induced during the AS regeneration process (Figure 6A). In contrast, expression of *PtoWOX1a* and *1b* was slightly down-regulated in AS regeneration. This down-regulation of *PtoWOX1s* may be a technical artifact as both *PtoWOX1s* were expressed specifically in leaves, thus their expression in the B1 stage may be still at a high level. Nevertheless, we view this as an indication that *PtoWOX1s* are not involved in SAM and AS regeneration. Finally, transcription of *PtoWOX2a*, *PtoWOX2b*, *PtoWOX11/12a*, and *PtoWOX11/12b*, whose expression was mainly in the roots, was unchanged during AS regeneration.

AR regeneration is composed of a R1 pre-induction stage (Additional file 1: Figure S4G), R2 callus formation stage (Additional file 1: Figure S4H), R3 AR emergence stage (Additional file 1: Figure S4I), and a R4 AR elongation stage (Additional file 1: Figure S4J). We examined the dynamics of *PtoWOXs* expression to gain insights into their possible roles in these processes. Expression of 14 out of 18 *PtoWOXs* was detected and changed during the AR regeneration process (Figure 6B). Among these, expression of four *WOX* genes: *PtoWOX5a, PtoWOX5b, PtoWOX11/12a*, and *PtoWOX11/12b* changed significantly during AR regeneration (Figure 6B). The expression of *PtoWOX11/12a* was over 80-fold stronger and maintained a high level in the R2 though R4 stages. *PtoWOX11/12b* was induced at the R3 and R4 stages, but not at the R1 or R2 stage. It is interesting that although expression of *PtoWOX5a* and *PtoWOX5b* was induced at the R2 stage, their expression gradually decreased in the following stages. Similarly, root specific *WOXs* were not induced in AS regeneration; the expression levels of *PtoWOX1a* and *PtoWOX1b*, two leaf-specific *WOX* genes in *P. tomentosa* (Figure 4), were not changed.

### Ectopic expression of *PtoWOX5a* and *PtoWOX11/12a* as along with *PtoWUSa* and *PtoWOX4a* promotes AR regeneration

**Figure 7:**
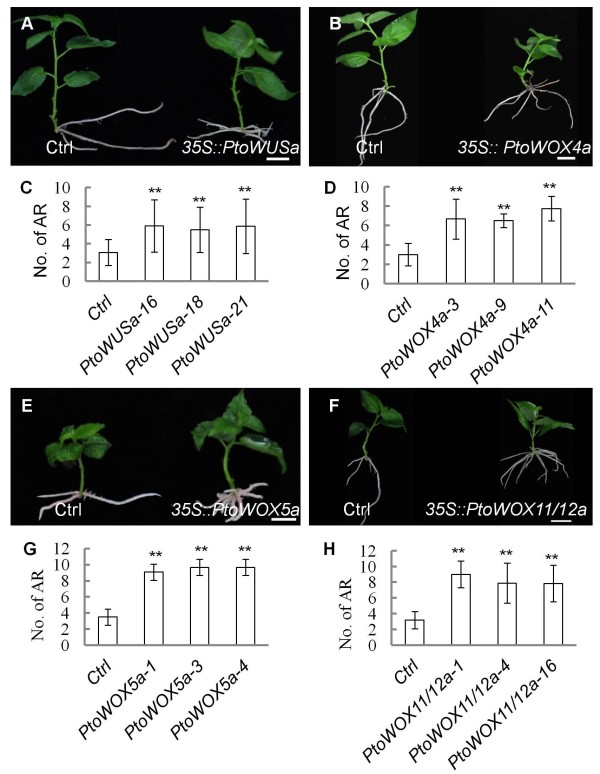
**Ectopic over-expression of four *PtoWOXs *promotes regeneration of AR. (A–B, E, and F)** Regenerated ARs in transgenic poplar plants ectopically expressing **(A)***PtoWUSa*, **(B)***PtoWOX4a*, **(E)***PtoWOX5a*, and **(F)***PtoWOX11/12a*. Wild type and over-expressing lines are on the left and right of each panel, respectively. **(C–D, G, and H)** Statistical result of ARs in different transgenic lines. No. of AR: adventitious root numbers; bars in **A**, **B**, **E**, and **F** represent 1 cm. ** in **C**, **D**, **G**, and **H** indicates significant difference using LSD test at *P* ≤0.01.

Because of the high expression of *PtoWOX5s*, *PtoWOX11/12 s*, *PtoWUSa*, and *PtoWOX4a* in the roots, and the strong induction of *PtoWOX5s* and *PtoWOX11/12 s* in the regeneration of AR, we were interested in the roles they play in the AR regeneration process. We used a 35S promoter to ectopically express *PtoWOX5a* and *PtoWOX11/12a* along with *PtoWUSa* and *PtoWOX4a* in the hybrid poplar (*P. alba* X *P. glandulosa*). AR regenerated in stem segments of three independent transgenic plants were quantified at the young sapling stage. All four ectopically expressed *WOX* genes promoted AR regeneration in the transgenic hybrid poplar lines (Figure 7). It appeared that *PtoWOX5a* and *PtoWOX11/12a* had a better ability than *PtoWUSa* and PtoWOX4a to promote the regeneration of AR. This is consistent with the strong induction of *PtoWOX5a* and *PtoWOX11/12a* in the regeneration of AR. Although the expression of *PtoWUSa* and *PtoWOX4a* was not obviously induced (Figure 6B), their effect on the regeneration of ARs when ectopically expressed indicated that the four *PtoWOX* genes may have overlapping functions.

## Discussion

### Diversification of *WOX* genes in *P. trichocarpa* and *P. tomentosa*

We identified 18 WOX encoding genes in both *P. trichocarpa* and *P. tomentosa*. Although these poplar *WOX genes* could be categorized into modern/WUS, intermediate, and ancient clades, it is interesting that no *WOX* genes from *P. trichocarpa* and *P. tomentosa* are classified together with *AtWOX3*, *AtWOX6*, *AtWOX7*, *AtWOX10*, or *AtWOX14*. Furthermore, many of the *WOX genes* in *P. trichocarpa* had sister copies owing to chromosomal duplication events.

While the roles of *AtWOX6* and *AtWOX7* are not well defined, *AtWOX3* is expressed in leaves and involved in leaf blade outgrowth [15,37]. Moreover, the function of the AtWOX3 protein can be fully complemented by AtWUS [15], and partially complemented by AtWOX4 [17]. It is possible that the WOX3-mediated function of leaf blade outgrowth regulation is compensated by other modern WOX members in poplars. In this regard, we note that the *PtoWOX1a* gene and *PtoWOX1b* gene are specifically expressed in *P. tomentosa* leaves (Figure 4).

*Arabidopsis* contains three ancient *WOX* genes, *AtWOX10*, *AtWOX13*, and *AtWOX14. P. trichocarpa* and *P. tomentosa* also have three ancient *WOX* genes, *PtrWOX13a, b*, and *c*; these are very similar in sequence, with *PtrWOX13b* and *PtrWOX13c* being sister pairs. Grape also has three ancient *WOX genes*, *VvWOX13A*, *B,* and *C*. [28]. There appears to be functional diversification in *AtWOX10*, *AtWOX13*, and *AtWOX14*; the *AtWOX13* gene is involved in replum formation during fruit development [21], *AtWOX14* acts redundantly with *AtWOX4* in vascular cell division [23], while the role of *AtWOX10* is unknown. The three ancient *WOX* genes of *P. trichocarpa* and *P. tomentosa* are ubiquitously expressed in all examined tissue in poplars (Figure 4). It is possible that the diversified functions of ancient *AtWOX10*, *13*, and *14* in *Arabidopsis* may be maintained by three ancient *WOX* genes, namely *WOX13a, b*, and *c* in poplars.

### Expression of *PtoWOX* genes

Protein function can be specified in terms of temporally and spatially regulated gene expression. We note that some *PtoWOXs* genes are expressed differently from their *Arabidopsis* counterparts (Table 2). *AtWUS* is expressed specifically in the rib-meristem (RM) cells beneath the central zone of the SAM, and it maintains the stem cell population via a regulatory loop with *CLAVATA* genes in *Arabidopsis*[9,32]. In poplar, the expression of *PtoWUSa* is not limited to the SAM and is strongly induced in the regeneration of AS; it is also detected in roots, stem and leaves (Figure 4, Figure 5E–G), and particularly in the CAM zone of stems. A major difference between *Arabidopsis* and poplar is that the latter is a perennial plant with annual differentiation of vascular tissues from CAM. It seems that the *PtoWUSa* gene may also be involved in vascular tissue differentiation. In *Arabidopsis*, the *AtWOX4* gene is required for vascular differentiation from CAM cells [12,38]. Both *PtoWOX4a* and *PtoWOX4b* genes are strongly expressed in CAM cells and are strongly induced in AS regeneration, but not in AR regeneration (Figure 6). Therefore, differently from *PtoWUSa*, the function of the *PtoWOX4* gene in CAM cells may be maintained in poplars. It will be interesting to test whether *PtoWUSa* and *PtoWOX4* act differently in vascular tissue differentiation in poplars.

*AtWOX5* is a QC cell specific gene in *Arabidopsis*[11]. It seems that functional expansion also occurs for *PtoWOX5s*. In poplars, the *PtoWOX5a* gene is strongly expressed in a small region that resembles QC cells, but is also detectable in young leaves (Figure 4). Furthermore, *PtoWOX5a* expression is strongly induced in the regeneration of AR as well as in the AS. Therefore, we speculate that the function of *PtoWOX5a* and *PtoWOX5b* may have expanded from roots to leaves in poplars.

Although the expression pattern of *AtWOX11* in *Arabidopsis* has not been reported, expression of *OsWOX11* in rice is detected in cell division regions of both roots and shoots [20]. We note that expression of both *PtoWOX11/12 s* is restricted to a small area behind the root cap region that resembles the root QC in AR tips, and probably the pericycle (Figure 5D). Thus, the action of *PtoWOX11/12 s* may be restricted to root development in poplars. During the regeneration of AR, the expression levels of *PtoWOX11/12 s* and *PtoWOX5s* are all strongly induced; however, it is interesting that the expression of *PtoWOX11/12a* is induced at the R2 stage, while the expression of *PtoWOX11/12b* is induced at the R3 stage. It is likely that *PtoWOX11/12a* acts differently from *PtoWOX11/12b* in the regeneration of AR.

### Four PtoWOX proteins can perform similar functions in promoting AR regeneration

A key function of WOX proteins is to maintain the stem cell population in different tissues and organs [9,11,12,39]. Recently, it has been demonstrated [8] by using a *lam1* complementation screen of WOX proteins in *N. sylvestris* that modern members of WOX proteins may have acquired additional functions during evolution that lead to functional specificity of this clade in the regulation of leaf blade outgrowth. In this study, we found that when ectopically expressed, three members of the modern WOXs (PtoWOX4a, PtoWOX5a, and PtoWUSa) and a member of the intermediate WOXs (PtoWOX11/12a) promote AR regeneration (Figure 7). In our *promoter::GUS* based expression analysis, the *PtoWUSa*, *PtoWOX5a*, and *PtoWOX11/12a* genes are expressed in a small region in the root tips resembling QC cells, the *PtoWOX4a* gene however, was not expressed in restricted areas in the root tips. Therefore, it is likely that members of the modern and intermediate WOXs can perform similar functions in the regeneration of AR in poplars, even if the gene is not normally expressed in root tip cells.

There is a clear divergence in the amino acid sequences of the 15 *Arabidopsis* and 18 poplar WOX proteins, with only modern WOX proteins possessing a WUS-box domain. Lin *et al*. [8] demonstrated that the WUS-box is required for the regulation of leaf blade outgrowth. Many different WOXs act in different cells and tissues to maintain the stem cell population [9,11,12,39], thus a common action mechanism for WOX proteins in maintaining the stem cell niche has been proposed [8]. Our results add support to this viewpoint. The regeneration of AR relies on a balance between cell differentiation and renewal of stem cells in the RAM [11]. It is possible that PtoWOX5a and PtoWOX11/12a, whose genes are expressed and strongly induced in the regeneration of AR, are major players in maintaining the stem cell niche in root tips. However, the function of PtoWOX5a and PtoWOX11/12a in maintaining the stem cell niche in root tips may be partially overlapped by PtoWUSa or PtoWOX4a. When the latter two *PtoWOX* genes are ectopically expressed they promote the regeneration of AR. This functional overlapping may be attributed to a common mechanism for maintaining the stem cell niche. It is possible that a WOX domain other than the WUS-box is responsible for this common action. It will be interesting to examine which WOX domains have evolutionary significance in the function of WOXs in stem cell maintenance.

## Conclusions

This study represents a step forward in our understanding of the functions and mechanisms of 18 WOXs found in poplar, in particular, their possible roles in vascular as well as root development of poplars. Based on the expression of *PtoWOX* genes revealed in this study, it is attempted to suggest that, in poplars, PtoWOX4s and PtoWUSs are involved in vascular development, while PtoWOX5s and ProWOX11/12s are major players in root development. Despite these possible functional specificities, it appears that there is a common action mechanism for different PtoWOXs in maintaining different stem cell niches.

## Methods

### Bioinformatic analysis

The *P. trichocarpa* genome (release 3.0, http://www.phytozome.net/poplar) was blasted using AtWOXs protein sequences as queries with NCBI BLASTP. Obtained sequences were used as secondary queries to re-blast the *P. trichocarpa* genome. After removing redundant sequences, multiple alignments of full length sequences of AtWOX and PtrWOX proteins were performed using the Clustal X2.0 program [40]. Maximum likelihood (ML) phylogenetic trees were constructed using PhyML (v3.0) with JTT amino acid substitution model, 1000 bootstrap replicates, estimated proportions of invariable sites, estimated gamma distribution parameters, and an optimized starting BIONJ tree [41,42]. A multiple alignment of full-length WOX protein sequences from *P. trichocarpa* and *P. tomentosa* was executed using Clustal X2.0 [40], and a phylogenetic tree constructed using MEGA 4.0 by the neighbor-joining (NJ) method with 1000 bootstrap replicates. The trees have been submitted to Treebase under study number 15612 (http://treebase.org/treebase-web/search/study/summary.html?id=15612). Accession numbers used in this study are listed in Additional file 2: Table S4. Exon and intron structures of individual *PtrWOXs* were illustrated using the Gene Structure Display Server (GSDS, http://gsds.cbi.pku.edu.cn/) [43] by aligning the cDNA sequences with the corresponding genomic DNA sequences from http://www.phytozome.net. All 18 *PtrWOX* genes were mapped to *P. trichocarpa* chromosomes. Whole-genome duplication analyses were accomplished as described in Tuskan *et al.*[30]. The MEME program (version 4.3.0, http://meme.sdsc.edu) [44] was used for elucidation of motifs in HDs. MEME was run locally with the following parameters: number of repetitions - any; maximum number of motifs - 20; and the optimum motif widths were constrained from 6 to 21 residues.

### Plasmids and constructs

The coding sequences of all *PtoWOXs* except for *PtoWUSb* (submitted to NCBI by another group) were amplified from the cDNA of *P. tomentosa*, and cloned into pDNOR222.1 (Life technologies, Carlsbad, California, U.S.) (to produce *pENTRs*) for sequencing. *PtoWUSa, PtoWOX4a, PtoWOX5a, and PtoWOX11/12a* were subcloned into pMDC32 to produce *35S::PtoWUSa*, *35S::PtoWOX4a*, *35S::PtoWOX5a,* and *35S::PtoWOX11/12a* constructs. The resultant *pENTR* constructs were then recombined into pEarleyGate104 (ABRC stock DB3-686) to produce *35S::YFP-PtoWUSa, 35S::YFP-PtoWOX4a, 35S::YFP-PtoWOX5a*, and *35S::YFP-PtoWOX11/12a* constructs using the Gateway cloning system (Life technologies, Carlsbad, California, U.S.). *PtoWOX13c* without the stop codon was amplified and subcloned into pEarleyGate101 (ABRC stock DB3-683) to produce *35S::PtoWOX13c-YFP.* The primer sequences used for amplification of *PtoWOXs* are listed in Additional file 2: Table S1. 5′UTR fragments, 2 ~ 3 kb in size, of *PtoWUSa, PtoWOX4a, PtoWOX5a,* and *PtoWOX11/12a* were amplified from the genomic DNA of *P. tomentosa* Carr. Primer sequences and promoter lengths are listed in Additional file 2: Table S1. The amplified promoter fragments were cloned into pDNOR222.1, and then subcloned into pMDC164 to produce *P*_*PtoWUSa*_*::GUS*, *P*_*PtoWOX4a*_*::GUS*, *P*_*PtoWOX5a*_*::GUS*, and *P*_*PtoWOX11/12a*_*::GUS* constructs using the gateway cloning system (Life technologies, Carlsbad, California, U.S.). At least three independent lines were used for analysis.

### Plant cultivation and transformation

Tobacco plants (*Nicotiana benthamiana*) used for transient expression were grown on soil under an 8/16 h (day/night) photoperiod at 20°C. All constructs were introduced into *Agrobacterium tumefaciens* strain GV3101 by electroporation. A single *A. tumefaciens* colony containing *35S::YFP-PtoWUSa*, *35S::YFP-PtoWOX4a*, *35S::YFP-PtoWOX5a, 35S::YFP-PtoWOX11/12a*, or *35S::PtoWOX13c-YFP* was used to inoculate 2 mL of YEP medium (per liter: 10 g tryptone, 10 g yeast extract, 5 g NaCl, pH 7.0), supplemented with 50 mg/L kanamycin, 10 mg/L gentamycin, and 34 mg/L rifampicin. Bacterial cultures were incubated at 28°C with agitation until OD_600_ = 0.5. 0.5 mL of culture was transferred into an Eppendorf tube, and the bacteria pelleted by centrifugation at 2000 × *g* for 5 min in a microcentrifuge at room temperature. The pellet was washed twice with 0.5 mL of infiltration buffer (10 mM MgCl_2_, 150 μM acetosyringone) and resuspended in 0.5 mL of the same buffer. The inoculum concentration of *35S::YFP-PtoWUSa*, *35S::YFP-PtoWOX4a*, *35S::YF-PtoWOX5a, 35S::YFP–PtoWOX11/12a*, and *35S::PtoWOX13c-YFP* (OD_600_ = 0.1) was adjusted by diluting the bacterial suspension with the infiltration buffer. The inoculum was delivered to tobacco lower epidermal leaf cells by gentle pressure infiltration using a 1-mL syringe without a needle. Following infiltration, plants were incubated under dark conditions for 6 h, and then grown under normal conditions, as described above. Tobacco leaves were analyzed 3 days after infiltration.

Hybrid poplar (*P. alba* X *P. glandulosa*) clone 84K used for transformation were kept at 23–25°C under a 16/8 h (day/night) photoperiod, with light intensity of 50 μM m^−2^ s^−1^ provided by cool white fluorescent tubes. Leaf-discs from 84K were infected with *Agrobacterium* cultures containing *P*_*PtoWUSa*_*::GUS*, *P*_*PtoWOX4a*_*::GUS*, *P*_*PtoWOX5a*_*::GUS*, *P*_*PtoWOX11/12a*_*::GUS*, *35S::PtoWUSa*, *35S::PtoWOX4a*, *35S::PtoWOX5a*, and *35S:: PtoWOX11/12a* constructs with OD_600_ = 0.3–0.8. Infected leaf-discs were co-cultured with *Agrobacteria* in the AS induction medium [SIM; Murashige-Skoog (MS) basal medium with 0.5 mg/l 6-benzyl aminopurine (6-BA) and 0.05 mg/l naphthaleneacetic acid (NAA)] in the dark for 3 days at 22 ± 2°C. Leaf-discs were transferred on SIM with 3 mg/L hygromycin and 200 mg/L timentin under a 16 h/8 h (light/dark) regime. After one month, individual regenerated shoots were removed and transferred onto root induction medium (RIM, 1/2 MS medium supplemented with 0.05 mg/L IBA, 0.02 mg/L NAA) containing 3 mg/L hygromycin and 200 mg/L timentin for AR induction. For AS induction, leaf discs were cultured on SIM for 18 days (Additional file 1: Figure S6A–F), while stems were cultured on RIM for 9 days for AR regeneration (Additional file 1: Figure S6G–J). At least three independently transformed lines were used for overexpression analysis and *promoter::GUS* assay analysis. At least 25 clones of each overexpressed line were used for AR regeneration. All experiments were repeated at least three times with similar results. The regeneration of AS and AR in *P. tomentosa* was performed as above, but without the *Agrobacteria* co-cultured procedure.

### RNA isolation, RT-PCR, qRT-PCR, and RNA-seq analysis

Total RNAs were extracted from roots, leaves, stem, CAM zone, and immature xylem of *P. tomentosa* Carr, and from materials at different stages and vegetative tissues of the hybrid poplar *P. alba X P. glandulosa* for RNA-sequencing using an RNeasy Plant Mini Kit and RNase-free DNase I set (Qiagen, Hilden, Germany). First-strand cDNA synthesis was carried out with approximately 1.5 μg RNA using the SuperScript III first-strand synthesis system (Life technologies, Carlsbad, California, U.S.) according to the manufacturer’s instructions. Specific RT-PCR primers were designed to have melting temperatures of 58–60°C and amplicon lengths of 150–260 bp using Primer3 software (http://frodo.wi.mit.edu/primer3/input.htm). The amplified fragments were separated on agarose gel electrophoresis. Real-time qRT-PCR was performed in quadruplicate using the SYBR Premix Ex Taq™ II Kit (TaKaRa Dalian, Dalian, China) on a Roche lightCycler 480 (Roche Applied Science, Penzberg, Upper bavaria, Germany) according to the manufacturer’s instructions. Quantification was performed using Lightcycler 480 software (Version 1.5.1.62, Roche). Expression was normalized relative to the control (Actin) using Roche LightCycler advanced relative quantification analysis (E-method, http://www.roche-applied-science.com/shop/products/gene-quantification-on-the-lightcycler-480-system) and fold changes (relatively to B1 or R1 stage) are shown in Figure 6. All experiments were repeated at least three times with similar results.

### *Promoter::GUS* assay

Histochemical GUS staining was performed as follows: 2-week old seedlings and 4-week old stem sections were first fixed in 90% cold acetone. Each sample was washed three times on ice using GUS staining buffer containing 50 mM sodium phosphate (pH7.0), 2 mM potassium ferrocyanide, 2 mM potassium ferricyanide, 10 mM EDTA, and 0.2% (v/v) Triton X-100. Fixed samples were transferred into the staining solution [GUS staining buffer with 20% (v/v) methanol, and 1 mM X-Gluc] and slowly vacuumed. After 12 h incubation at 37°C with gentle agitation, samples were rinsed in 70% ethanol for imaging. At least five clones for every *promoter::GUS* line were used for GUS staining. All experiments were repeated at least three times with similar results.

### Protein localization analysis

Tobacco leaf samples were analyzed 48 to 72 h after infiltration. Fluorescence of plant epidermal cells was observed using a LSM 510 confocal laser scanning microscope (Carl Zeiss AG, Oberkochen, Germany). Detection of fluorescence was performed as follows: fluorescence imaging of YFP excitation at 514 nm; scanning at 520–555 nm.

## Availability of supporting data

The phylogenetic trees generated in this study are available in Treebase under study number 15612 (http://treebase.org/treebase-web/search/study/summary.html?id=15612).

## Competing interest

The authors declare that they have no competing interests.

## Authors’ contributions

BL carried out all the constructions, transformation and data collection. LW performed most of the quantitative RT-PCR and data analysis. JZ and JL helped in poplar materials collection and total RNA extraction. HZ helped in experiment design, data interpretation and manuscript preparation. ML and JC conceived the project, supervised the analysis and critically revised the manuscript. All authors read and approved the final manuscript.

## Supplementary Material

Additional file 1: Figure S1. Sequence comparison of poplar WOX family proteins. A. Alignment of the HD sequences. Asterisks indicate residues that are highly conserved in HDs. B. Alignment of the WUS box that is located downstream of HDs. Note that no WUS box was found in PtrWOX13, 9, and 11/12 sub-classes. C. Alignment of the EAR-like domains from WUS and WOX5 proteins. **Figure S2.** Chromosomal location of *PtrWOX* genes. The schematic diagram of 18 *PtrWOX* genes in 14 chromosomes. Homologous blocks derived from the segmental duplication are indicated using the same colors. The diagram of the genome-wide chromosome organization resulting from genome duplication events in *P. trichocarpa* is adapted from Tuskan *et al*. [31]. **Figure S3.** Expression analysis of *PagWOX* genes in different tissues. A. Heat map of hierarchical clustering of *PagWOX* genes in vegetative tissues (YL, young leaves; ML, mature leaves; PS, primary stem; SS, secondary stem; R, roots). The data was obtained from our unpublished RNA-seq data. The expression level of genes was determined based on the value of RPKM (reads per kilobase of exon region in a gene per million mapped reads). The clustering was made on expression pattern. Details of the RPKM are shown in Table S3. Color scale represents log2 expression values. **Figure S4.** The stages of AS and AR regeneration. A-F Leaf explants in indicated stages of AS regeneration. B1-B6 represent the status of explants on 0, 6, 9, 12, 15, 18 days after AS induction. G-J Stem segments in indicated stages of AR regeneration. R1-R4 represent the stem status on 0, 3, 6, 9 days after AR induction. Bar represents 1 cm in A-J. Click here for file

Additional file 2: Table S1. Primer sequences for amplification of *PtoWOX*s and promoters and qPCR analysis. **Table S2.** Similarity of WOX sequences from *P. trichocarpa* and *P. tomentosa*. **Table S3.** RPKM value of *PagWOX* genes in vegetative tissues obtained from RNA-seq data. **Table S4.** Accession number of proteins analyzed in this study. Click here for file
