# Supplementary figures and images for: WUSCHEL-related Homeobox genes in Populus tomentosa: diversified expression patterns and a functional similarity in adventitious root formation
Source: BMC Genomics. 2014 Apr 21;15:296. doi: 10.1186/1471-2164-15-296 (PMC4023605; doi:10.1186/1471-2164-15-296)

Figure S1

## A Homedomain

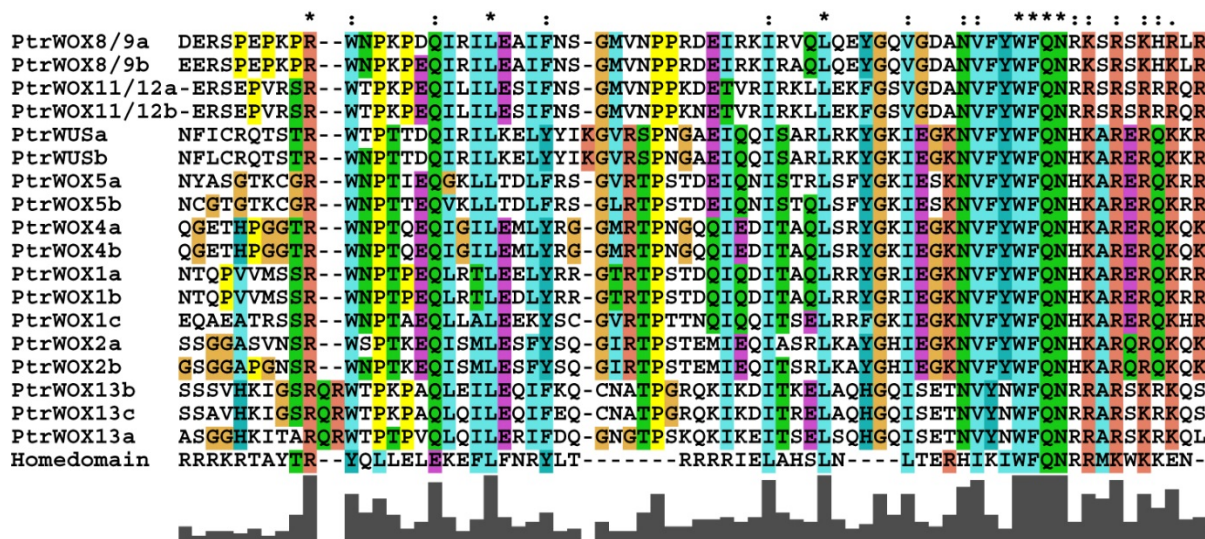

## B WUS Box

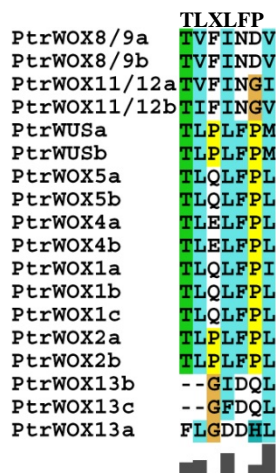

## C EAR-like domain

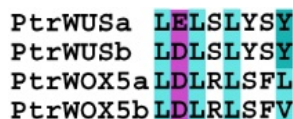

Figure S2

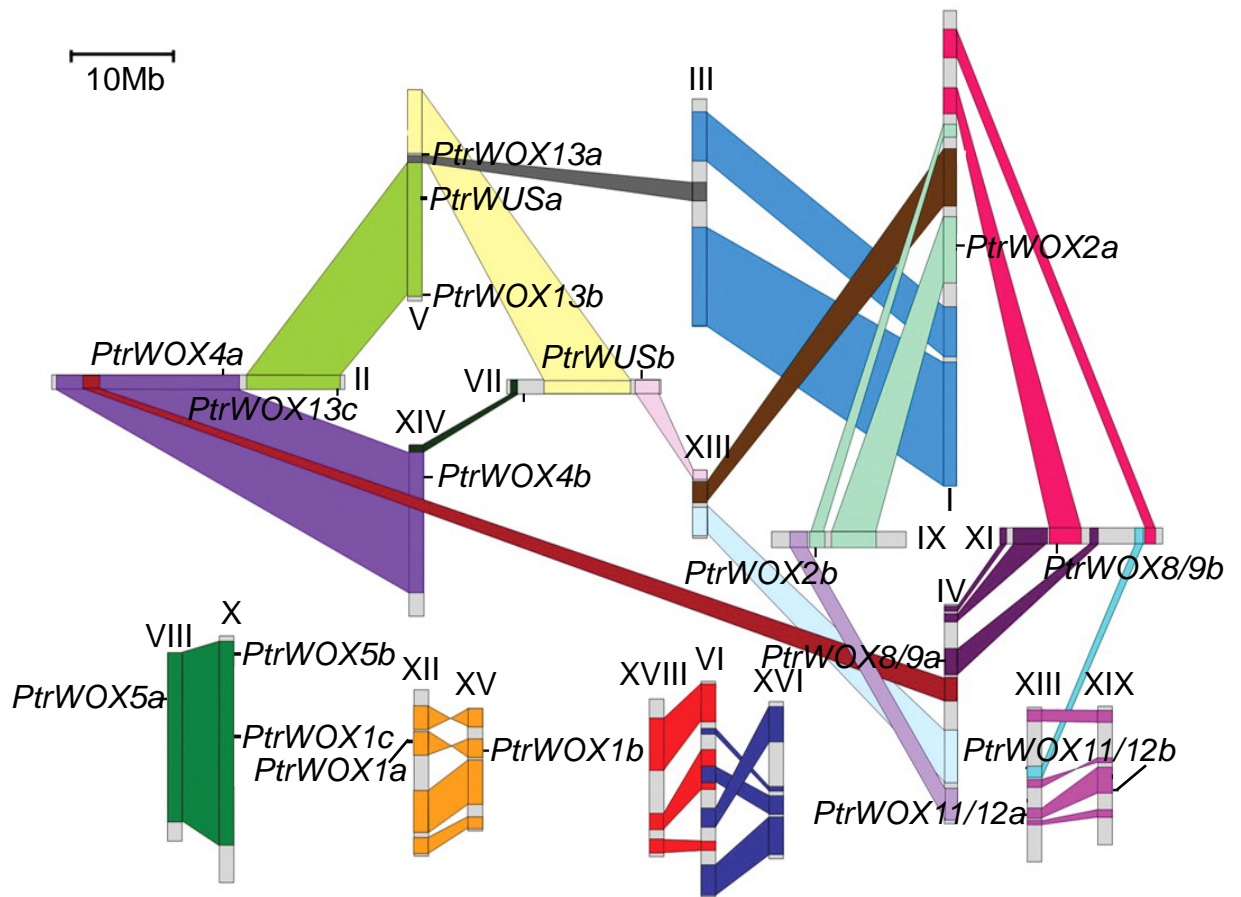

Figure S3

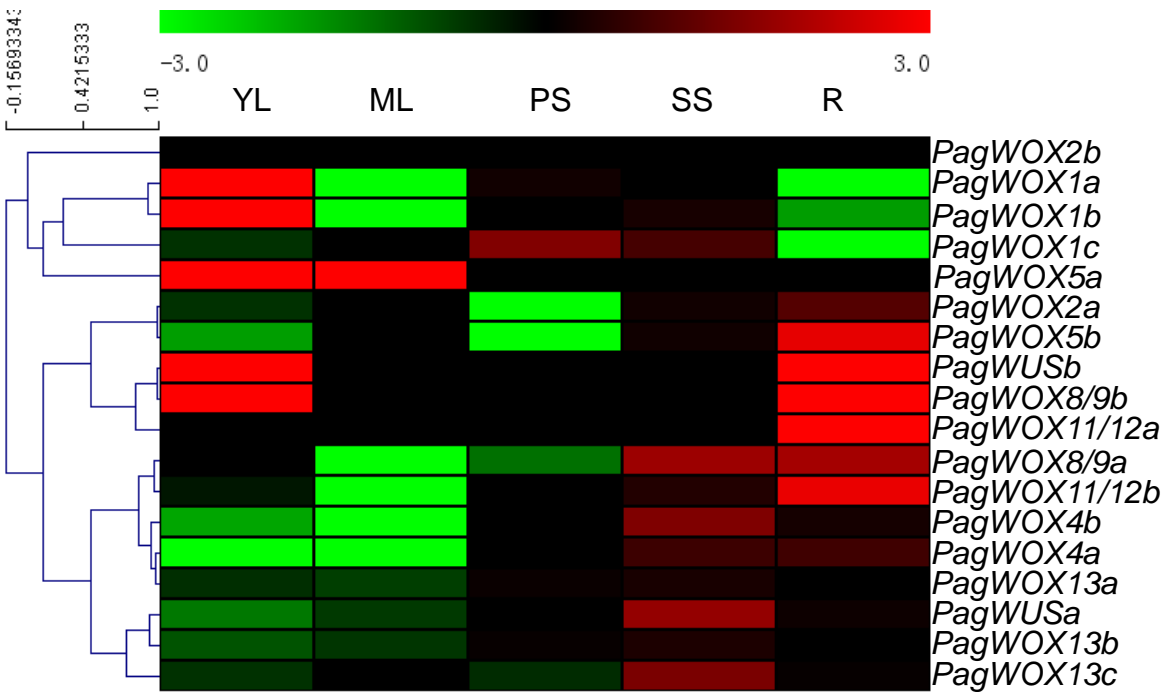

Figure S4

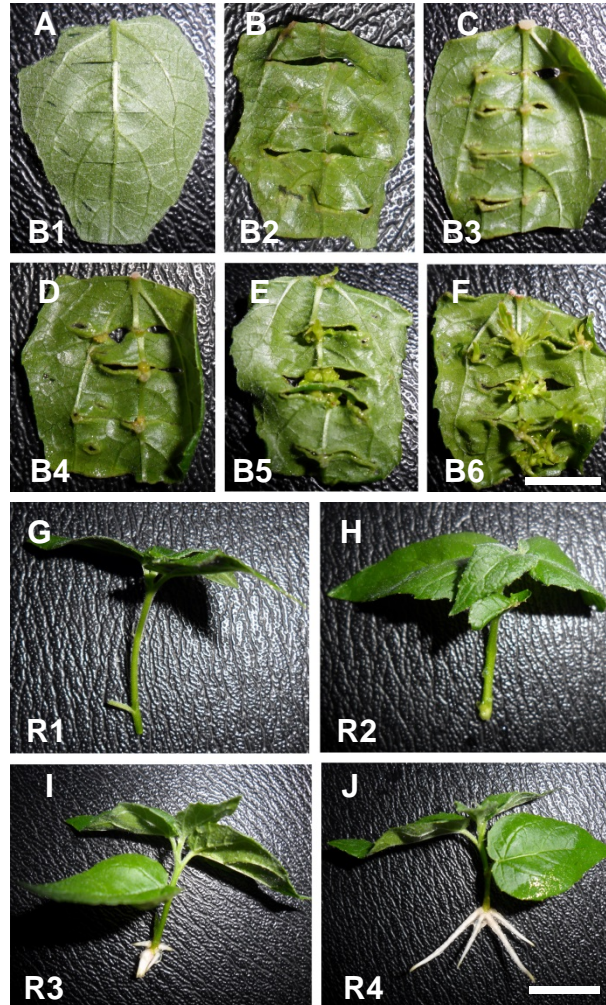

Supplement: Additional file 1: Figure S1. — Sequence comparison of poplar WOX family proteins. A. Alignment of the HD sequences. Asterisks indicate residues that are highly conserved in HDs. B. Alignment of the WUS box that is located downstream of HDs. Note that no WUS box was found in PtrWOX13, 9, and 11/12 sub-classes. C. Alignment of the EAR-like domains from WUS and WOX5 proteins. Figure S2. Chromosomal location of PtrWOX genes. The schematic diagram of 18 PtrWOX genes in 14 chromosomes. Homologous blocks derived from the segmental duplication are indicated using the same colors. The diagram of the genome-wide chromosome organization resulting from genome duplication events in P. trichocarpa is adapted from Tuskan et al. [31]. Figure S3. Expression analysis of PagWOX genes in different tissues. A. Heat map of hierarchical clustering of PagWOX genes in vegetative tissues (YL, young leaves; ML, mature leaves; PS, primary stem; SS, secondary stem; R, roots). The data was obtained from our unpublished RNA-seq data. The expression level of genes was determined based on the value of RPKM (reads per kilobase of exon region in a gene per million mapped reads). The clustering was made on expression pattern. Details of the RPKM are shown in Table S3. Color scale represents log2 expression values. Figure S4. The stages of AS and AR regeneration. A-F Leaf explants in indicated stages of AS regeneration. B1-B6 represent the status of explants on 0, 6, 9, 12, 15, 18 days after AS induction. G-J Stem segments in indicated stages of AR regeneration. R1-R4 represent the stem status on 0, 3, 6, 9 days after AR induction. Bar represents 1 cm in A-J. [file 1471-2164-15-296-S1.pdf]
