# Supplementary material for: WUSCHEL-related Homeobox genes in Populus tomentosa: diversified expression patterns and a functional similarity in adventitious root formation
Source: BMC Genomics. 2014 Apr 21;15:296. doi: 10.1186/1471-2164-15-296 (PMC4023605; doi:10.1186/1471-2164-15-296)
Supplement: Additional file 2: Table S1. — Primer sequences for amplification of PtoWOXs and promoters and qPCR analysis. Table S2. Similarity of WOX sequences from P. trichocarpa and P. tomentosa. Table S3. RPKM value of PagWOX genes in vegetative tissues obtained from RNA-seq data. Table S4. Accession number of proteins analyzed in this study. [file 1471-2164-15-296-S2.pdf]

Supplementary tables

Table S1. Primer sequences for amplification of *PtoWOX*s and promoters and qPCR analysis.

Primer	Primer sequence	Application
PtoWUSaF	GGGGACAACTTTGTACAAAAAAGTTGGAATGGAACCTCAACAGCAACAAC	cloning
PtoWUSaR	GGCGGCCGCACAACTTTGTACAAGAAAGTTGGGTATTAATAATCTTGTTGCTGGCCATT	
PtoWOX1aF	GGGGACAACTTTGTACAAAAAAGTTGGAATGTGGATGATGGGTTACAATGATA	
PtoWOX1aR	GGCGGCCGCACAACTTTGTACAAGAAAGTTGGGTATTAGATCCTCAGTGGAAGGAACTCG	
PtoWOX1bF	GGGGACAACTTTGTACAAAAAAGTTGGAATGTGGATGATGGGTTATAATGATG	
PtoWOX1bR	GGCGGCCGCACAACTTTGTACAAGAAAGTTGGGTATTAGTGCTTCAGTGGAAGGAACTC	
PtoWOX2aF	GGGGACAACTTTGTACAAAAAAGTTGGAATGGACGTGTCTAGTTCTGGTGG	
PtoWOX2aR	GGCGGCCGCACAACTTTGTACAAGAAAGTTGGGTATCAATGAGAACTCTCGAAAGGATCT	
PtoWOX2bF	GGGGACAACTTTGTACAAAAAAGTTGGAATGGATAGTGATGATATGGACGTGG	
PtoWOX2bR	GGCGGCCGCACAACTTTGTACAAGAAAGTTGGGTATTAAGAATCTTTTCCATAGAAGAAATCA	
PtoWOX4a/bF	GGGGACAACTTTGTACAAAAAAGTTGGAATGGATGGGAAGCATGAAGGTGCA	
PtoWOX4aR	GGCGGCCGCACAACTTTGTACAAGAAAGTTGGGTATCATCTGCCTTCCGGGTGCA	
PtoWOX4bR	GGCGGCCGCACAACTTTGTACAAGAAAGTTGGGTATCATCTTCCTTCCGGGTGC	
PtoWOX5aF	GGGGACAACTTTGTACAAAAAAGTTGGAGTACGCATCTGAAGCTAACAGAGAC	
PtoWOX5aR	GGCGGCCGCACAACTTTGTACAAGAAAGTTGGGTAGATTCGAAGCTTGTTTTACAGAAAG	
PtoWOX5bF	GGGGACAACTTTGTACAAAAAAGTTGGAATGGAAGAGAGAATGTCAGGCTTT	

PtoWOX5bR	GGCGGCCGCACAACTTTGTACAAGAAA<br>GTTGGGTACTACACAAAGCTTAATCGCA<br>GATCT	
PtoWOX1cF	GGGGACAACTTTGTACAAAAAAGTTGG<br>AATGTGGATGATAAATGGTGGTGAC	
PtoWOX1cR	GGCGGCCGCACAACTTTGTACAAGAAA<br>GTTGGGTATCAGTTCTTCAGAGGAAGAA<br>ACTCA	
PtoWOX8/9aF	GGGGACAACTTTGTACAAAAAAGTTGG<br>AATGGCTTCATCAAACAAACACTG	
PtoWOX8/9aR	GGCGGCCGCACAACTTTGTACAAGAAA<br>GTTGGGTATTATATATGTTCGCTCATGG<br>AA	
PtoWOX8/9bF	GGGGACAACTTTGTACAAAAAAGTTGG<br>AATGGCTTCATCAAACAGACACTG	
PtoWOX8/9bR	GGCGGCCGCACAACTTTGTACAAGAAA<br>GTTGGGTACTATATATGTTCACTAATGG<br>AAAGGGGT	
PtoWOX11/12a/bF	GGGGACAACTTTGTACAAAAAAGTTGG<br>AATGGAAGATAATCAAGGCCAAGAC	
PtoWOX11/12aR	GGCGGCCGCACAACTTTGTACAAGAAA<br>GTTGGGTATTATGCTCCAGAGATGATTA<br>CCAG	
PtoWOX11/12bR	GGCGGCCGCACAACTTTGTACAAGAAA<br>GTTGGGTATTATACTGCAGAGATGTTTA<br>CCAGG	
PtoWOX13aF	GGGGACAACTTTGTACAAAAAAGTTGG<br>AATGGACTGGGACAACAATCAAGA	
PtoWOX13aR	GGCGGCCGCACAACTTTGTACAAGAAA<br>GTTGGGTATCAGCCTGCCATGCCATAGT	
PtoWOX13bF	GGGGACAACTTTGTACAAAAAAGTTGG<br>AATGGAGGATGGAAAGTTTCAAAAT	
PtoWOX13bR	GGCGGCCGCACAACTTTGTACAAGAAA<br>GTTGGGTATCATCCAAACAAGTCATATT<br>GCTCC	
PtoWOX13cF	GGGGACAACTTTGTACAAAAAAGTTGG<br>AATGGAGGAGGGGAGGTTTCA	
proPtoWUSaF	GGGGACAACTTTGTACAAAAAAGTTGG<br>ACGTGGGATTGAACTTTCCCTG	cloning<br>2328 bp
proPtoWUSaR	GGCGGCCGCACAACTTTGTACAAGAAA<br>GTTGGGTAGATGGATTGAGAAATCAGA<br>AAAAAA	
proPtoWOX4aF	GGGGACAACTTTGTACAAAAAAGTTGG<br>AAACCTTTACGTGTTGTTCTCTTTTG	cloning

proPtoWOX4aR	GGCGGCCGCACAACTTTGTACAAGAAAGTTGGGTAGGCGAAGGACTGAATGATAGGG	3293 bp
proPtoWOX5aF	GGGGACAACTTTGTACAAAAAAGTTGGACTATGACCAGTTCTTTTTTCCCG	cloning 3006 bp
proPtoWOX5aR	GGCGGCCGCACAACTTTGTACAAGAAAGTTGGGTATGTCTCTGTTAGCTTCAGATGCGTA	
proPtoWOX11/12aF	GGGGACAACTTTGTACAAAAAAGTTGGATAGGCGTTGTGTGAAGGTTCG	cloning 3194 bp
proPtoWOX11/12aR	GGCGGCCGCACAACTTTGTACAAGAAAGTTGGGTATACTGTGAAATGAGGAAAATAAATGTTT	
PtoWUSaF	ACAATGGCCAGCAACAAGAT	qRT-PCR
PtoWUSaR	CAATAAACAAGTGCCCAGCA	
PtoWUSbF	ATTAATGTTTGTGAATATTATCCCT	
PtoWUSbR	AAGAAGCACATATATAAAATAGCAC	
PtoWOX1aF	AAGGAGCGGCAACAGTAAGA	
PtoWOX1aR	ATTCACCTCCCTCGTTGATG	
PtoWOX1bF	TGGCTGATCAAAACAAAAGAGA	
PtoWOX1bR	TACGAAGCTGTGCGGTTATG	
PtoWOX2aF	TCACGGTTCAACTTCTGCTG	
PtoWOX2aR	TCAATGAGAACTCTCGAAAGGA	
PtoWOX2bF	ATGGATAGTGATGATATGGACGTG	
PtoWOX2bR	GCCTGCTCGTTATTTGCTCT	
PtoWOX4aF	ATCTGCCTTGACGTGCTTTT	
PtoWOX4aR	AATCTTTTCTCAGCAAGGTTTCC	
PtoWOX4bF	TTCGAGAAAGAAGAAAGGCAAG	
PtoWOX4bR	GGTTTGTCTGAACTCTCAACAAGT	
PtoWOX5aF	TTTTCTTCTGCGCGATATTTT	
PtoWOX5aR	TTCTCTGACTCTACTTCATCAAAGGA	
PtoWOX5bF	CTTCTGCGCGATATTTTACTGA	
PtoWOX5bR	CCCGCTTCATCAAAGGAGT	
PtoWOX1c	TCAAAGAGCAAGCAGAAGCA	
PtoWOX1c	TCAATCTTCCCAAACCTTCG	
PtoWOX8/9aF	AAGAGTATGGACAAGTTGGGGAT	
PtoWOX8/9aR	GTCTGTTGAGAGTGAAAGAAAAA	
PtoWOX8/9bF	CATCGTCTCTATCATCTTCCTCA	
PtoWOX8/9bR	TAAAAGCCACAGTTTCCCCTCCA	
PtoWOX11/12aF	CTGGTTTCAAAACCGACGAT	
PtoWOX11/12aR	CGGGGATTGAACAAAAGAAG	
PtoWOX11/12bF	GACTCCAAAGCCAGAGCAAA	
PtoWOX11/12bR	ACCCATTAGAAGTGCCACCA	
PtoWOX13cF	GTGCCTGTCAAGAGGTAGGC	

PtoWOX13cR	GAGCCTTGTGCATCTCAACA
PtoWOX13bF	CGAGGTCTGAAGCAAGAACC
PtoWOX13bR	CAAAGGAAAGAGAGGCAACG
PtoWOX13aF	TGGACTGGGACAACAATCAA
PtoWOX13aR	TGGGCAGAGAGGGTTTTATG

Table S2. Sequence similarity of WOXs from *P. tricocarpa* and *P. tomentosa*

	Protein sequence identity/%	CDS sequence idrntity%
WUSa	98.86	98.99
WUSb	96.59	97.23
WOX1a	94.06	95.79
WOX1b	99.2	99.29
WOX2a	96.75	97.84
WOX2b	96.75	97.57
WOX4a	98.12	98.91
WOX4b	99.53	99.69
WOX5a	96.13	97.99
WOX5b	91.71	93.22
WOX1c	95.89	97.79
WOX8/9a	94.62	95.74
WOX8/9b	97.1	97.89
WOX11/12a	97.65	98.57
WOX11/12b	86.75	87.87
WOX13a	99.6	99.33
WOX13b	97.69	98.31
WOX13c	98.62	97.71

Table S3. RPKM value of *PagWOX* genes obtained from RNA-seq in vegetative tissues* of a hybrid poplar *P. alba X P. glandulosa* .

	**YL**	**ML**	**PS**	**SS**	**R**
PagWUSa	0.03	0.05	0.08	0.27	0.09
PagWUSb	0.04	0.001	0.001	0.001	0.23
PagWOX1a	21.92	0.001	0.14	0.12	0.001
PagWOX1b	11.95	0.001	0.18	0.22	0.05
PagWOX2a	0.04	0.06	0.001	0.07	0.12
PagWOX2b	0.001	0.001	0.001	0.001	0.001
PagWOX1c	0.06	0.09	0.26	0.16	0.001
PagWOX4a	3.74	1.69	33.01	54.56	55.76
PagWOX4b	16.23	1.02	63.17	179.98	75.73
PagWOX5a	0.06	0.09	0.001	0.001	0.001
PagWOX5b	0.11	0.4	0.001	0.46	2.65
PagWOX8/9a	0.15	0.001	0.06	0.54	0.58
PagWOX8/9b	0.02	0.001	0.001	0.001	0.21
PagWOX11/12a	0.001	0.001	0.001	0.001	0.12
PagWOX11/12b	0.05	0.001	0.06	0.08	0.41
PagWOX13a	16.09	14.11	25.91	29.03	23.55
PagWOX13b	8.7	11.1	18.41	21.82	17.21
PagWOX13c	10.66	15.92	11.14	43.73	16.87

*Note: YL, young leaves; ML, mature leaves; PS, primary stem; SS, secondary stem; R, roots.

Table S4. Accession number of proteins analyzed in this research.

Protein name	Accession number	Protein name	Accession number
PtrWUSa	XM006383052	PtoWOX5a	KF982705
PtrWOX1a	XM002317841	PtoWOX5b	KF982706
PtrWOX1b	XM002322064	PtoWOX8/9a	KF982708
PtrWOX1c	XM002314766	PtoWOX8/9b	KF982709
PtrWOX2a	XM002298328	PtoWOX11/12a	KF982710
PtrWOX2b	XM002313406	PtoWOX11/12b	KF982711
PtrWOX4a	XM002301134	PtoWOX13a	KF982712
PtrWOX4b	XM002327131	PtoWOX13b	KF982713
PtrWOX5a	XM002312109	PtoWOX13c	KF982714
PtrWOX5b	XM002315137	PtoWUSb	FJ232064
PtrWOX8/9a	XM002305063	AtWUS	NM127349
PtrWOX8/9b	XM006377383	AtWOX1	AY251394
PtrWOX11/12a	XM002319161	AtWOX2	AY251392
PtrWOX11/12b	XM002325406	AtPRS/AtWOX3	AY251397
PtrWOX13a	XM002306396	AtWOX4	AY251396
PtrWOX13b	XM002306947	AtWOX5	AY251398
PtrWOX13c	XM002301881	AtWOX6	AY251399
PtrWUSb	XM002310270	AtWOX7	NM120659
PtoWUSa	KF982698	AtWOX8	AY251400
PtoWOX1a	KF982699	AtWOX9	AY251401
PtoWOX1b	KF982700	AtWOX10	NM101923
PtoWOX1c	KF982707	AtWOX11	AY251402
PtoWOX2a	KF982701	AtWOX12	AY251403
PtoWOX2b	KF982702	AtWOX13	AY251404
PtoWOX4a	KF982703	AtWOX14	NM101922
PtoWOX4b	KF982704		
